# Artificial Intelligence in Paediatric Allergy: From Diagnostic Support to Precision Medicine

**DOI:** 10.7759/cureus.112707

**Published:** 2026-07-15

**Authors:** Prav Hamal

**Affiliations:** 1 Paediatric Emergency Medicine, St. Mary's Hospital, London, GBR

**Keywords:** allergy, artifical intelligence, literature review, paediatrics, precision medicine

## Abstract

Paediatric allergic disease continues to increase globally, placing substantial pressure on healthcare systems and specialist allergy services. Current diagnostic approaches rely heavily on clinical history, skin prick testing, serum-specific immunoglobulin E (IgE), and oral food challenges, all of which have recognised limitations. Artificial intelligence (AI) has emerged as a promising tool capable of integrating complex data from structured electronic health records and unstructured notes to improve diagnostic accuracy, risk prediction, and healthcare delivery. This narrative review examined developments in AI applications within paediatric allergy between 2015 and 2025 and explored future opportunities and challenges for clinical implementation. A literature search of PubMed, MEDLINE, and Google Scholar was performed using combinations of the terms “paediatric allergy”, “food allergy”, “anaphylaxis”, “artificial intelligence”, “machine learning”, “deep learning”, and “clinical decision support”, with original research articles, systematic reviews, and relevant commentaries included. After screening 280 articles, 16 were included in the final narrative synthesis. Three principal themes emerged: diagnostic support and risk prediction, clinical decision support systems, and digital health technologies. Machine learning models integrating clinical history, biomarker profiles, and component-resolved diagnostics demonstrated improved prediction of food allergy outcomes compared with traditional approaches alone. Emerging applications include risk stratification for severe allergic reactions, support for drug allergy de-labelling pathways, and AI-assisted interpretation of oral food challenge outcomes. Recent advances in generative AI and large language models have additionally created opportunities for patient education, clinical documentation, and service efficiency. However, most studies remain retrospective and lack robust external validation. Overall, AI has significant potential to transform paediatric allergy through improved diagnostic precision, personalised risk prediction, and enhanced healthcare delivery. This review summarises current applications of AI in diagnosis, clinical decision support, digital health and future precision allergy medicine while highlighting key challenges to implementation.

## Introduction and background

Paediatric allergic disease has emerged as one of the most significant chronic health challenges affecting children worldwide. The prevalence of food allergy, atopic dermatitis, asthma, allergic rhinitis, and drug hypersensitivity has increased substantially over recent decades, particularly within high-income countries, although rising rates are increasingly being observed globally [1,2]. Food allergy alone is estimated to affect up to 8% of children in Western populations and represents a major cause of emergency department attendance and anaphylaxis-related hospitalisation [1,2]. Beyond its direct clinical consequences, allergic disease imposes substantial psychosocial, educational, and economic burdens on affected children and their families. Food avoidance, fear of accidental exposure, repeated healthcare encounters, and restrictions on social activities frequently contribute to reduced quality of life and increased parental anxiety [2].

Despite advances in allergy medicine, diagnosis and management remain inherently complex. Unlike many chronic conditions, allergy diagnosis rarely relies upon a single definitive investigation. Instead, clinicians must integrate clinical history with investigations such as serum-specific IgE testing, skin prick testing, component-resolved diagnostics, and oral food challenges [3,4]. However, sensitisation identified through skin prick testing or specific IgE measurement does not necessarily indicate clinically significant allergy, resulting in overdiagnosis, unnecessary dietary restriction, and avoidable specialist referral [2,3]. Conversely, oral food challenges remain the diagnostic gold standard but are resource-intensive, time-consuming, and associated with the risk of severe allergic reactions, limiting their widespread availability [4].

Unlike adult allergy, paediatric allergic disease is characterised by dynamic immunological maturation, evolving patterns of sensitisation, and the potential for spontaneous resolution of conditions such as cow's milk and egg allergy, making diagnosis and longitudinal risk prediction particularly challenging [4]. Furthermore, increasing allergy prevalence, rising demand for specialist services, and workforce shortages have created a pressing need for innovative technologies capable of improving diagnostic accuracy, supporting clinical decision-making, and enabling more efficient delivery of paediatric allergy care [2,3].

These challenges have coincided with increasing pressures on healthcare systems and specialist allergy services. Many healthcare systems continue to face shortages of paediatric allergists, resulting in prolonged waiting times, delayed diagnoses, and significant variation in access to specialist care [1,4]. Consequently, there is growing interest in technologies capable of improving diagnostic efficiency, supporting clinical decision-making, and optimising healthcare resource allocation [1,3,4].

Artificial intelligence refers to computational systems capable of performing tasks that traditionally require human intelligence, including pattern recognition, prediction, classification, and decision support [5]. Within healthcare, AI encompasses a range of methodologies including machine learning, deep learning, natural language processing, and, more recently, generative AI systems such as large language models (LLMs) [5-7]. Unlike conventional statistical approaches that analyse a limited number of variables, AI systems can integrate large multidimensional datasets and identify complex relationships that may not be readily apparent to clinicians [5,6]. Over the past decade, these technologies have demonstrated considerable promise across multiple medical specialities, including radiology, oncology, cardiology, dermatology, and emergency medicine [6,7].

Several characteristics make paediatric allergy particularly suited to AI applications. Allergy care routinely generates diverse datasets including clinical histories, biomarker profiles, skin prick test results, component-resolved diagnostics, environmental exposure information, treatment responses, and patient-reported outcomes [1,6]. Traditionally, these have been split into structured electronic health records (e.g., serum-specific IgE values) and unstructured data (clinical written notes, discharge summaries), which have been the basis of AI applications [2,3]. The increasing adoption of electronic health records and digital health technologies has further expanded the volume of available data [6]. Consequently, AI offers opportunities not only to improve diagnostic accuracy but also to identify novel disease phenotypes, predict disease progression, stratify anaphylaxis risk, and facilitate more personalised approaches to patient management [4,6,7].

Although AI has attracted considerable attention within healthcare, its application within paediatric allergy remains relatively underexplored compared with other specialities. Furthermore, recent advances in machine learning, digital health technologies, and generative AI have rapidly expanded the scope of potential applications. This review therefore examines developments in artificial intelligence within paediatric allergy between 2015 and 2025, focusing on diagnostic support, risk prediction, clinical decision support systems, digital health technologies, and future directions towards precision allergy medicine.

## Review

Literature search strategy

A narrative review of the literature was performed to evaluate current evidence regarding AI in paediatric allergy. Searches were conducted using PubMed, MEDLINE, and Google Scholar for studies published between January 2015 and March 2025.

Search terms included combinations of “paediatric allergy”, “food allergy”, “anaphylaxis”, “artificial intelligence”, “machine learning”, “deep learning”, “oral food challenge”, “clinical decision support”, and “digital health”.

Original research studies, systematic reviews, narrative reviews, and relevant expert commentaries published in English were included. Studies focusing exclusively on adult populations without relevance to paediatric allergy were excluded. As this was a narrative review, article selection was based on clinical relevance rather than formal systematic review methodology, and Preferred Reporting Items for Systematic Reviews and Meta-Analyses (PRISMA) reporting and risk-of-bias assessment were not undertaken.

Inclusion Criteria

The inclusion criteria were as follows: (i) Publications in English between January 2015 and March 2025; earlier landmark publications were also included where necessary to provide important clinical background; (ii) Studies investigating the application of AI, machine learning (ML), deep learning (DL), natural language processing (NLP), or generative AI in paediatric allergy or closely related allergy/immunology settings; (iii) Studies examining clinically relevant applications, including diagnostic support, risk prediction, clinical decision support, oral food challenge prediction, drug allergy assessment, digital health technologies, or healthcare workflow optimisation; (iv) Original research articles, systematic reviews, narrative reviews, and expert consensus or commentary articles that contributed substantially to the review objectives; (v) Studies including findings directly applicable to children or adolescents or providing evidence with clear relevance to paediatric allergy practice.

Exclusion Criteria

The exclusion criteria were as follows: (i) Studies focusing exclusively on adult populations without clear relevance to paediatric allergy; (ii) Studies examining AI applications unrelated to allergy or immunology; (iii) Conference abstracts, editorials, letters, study protocols, or duplicate publications that did not provide sufficient primary data or evidence synthesis; (iv) Studies describing theoretical AI methodologies without discussion of their clinical application; (v) Studies judged to provide limited additional information beyond that available in higher-quality or more contemporary publications addressing the same topic.

Results

Electronic database searches identified 280 records. Following the removal of duplicate and irrelevant publications, one reviewer (PH) screened 214 titles and abstracts. Sixty-one full-text articles were assessed for eligibility, of which 16 publications met the inclusion criteria for the final narrative synthesis. Additional landmark publications published before 2015 were included to provide essential background regarding allergy diagnosis and AI.

The literature was subsequently synthesised into three themes: diagnostic support and risk prediction, clinical decision support systems, and digital health and generative AI.

AI in Diagnosis and Risk Prediction

The strongest evidence for AI within paediatric allergy relates to diagnostic prediction. Traditional allergy investigations frequently demonstrate limited specificity when interpreted independently, contributing to overdiagnosis and unnecessary dietary restriction [3,4]. Consequently, machine learning models have increasingly been developed to integrate clinical history, demographic variables, serum-specific IgE levels, skin prick testing, and molecular allergology data.

Food allergy has been the principal focus of these developments. Recent predictive models have demonstrated improved performance in predicting oral food challenge outcomes compared with conventional diagnostic thresholds alone [8,9]. In peanut allergy specifically, machine learning algorithms have shown promise in reducing unnecessary oral food challenges while improving identification of clinically significant allergy [8,9].

AI has additionally been explored for risk stratification of severe allergic reactions. Predictive systems incorporating previous reaction severity, coexisting asthma, allergen exposure patterns, and biomarker profiles have demonstrated potential to identify children at increased risk of anaphylaxis and future severe reactions [10].

An emerging area involves AI-assisted interpretation of component-resolved diagnostics and molecular allergology. These technologies generate large quantities of highly granular data that may exceed routine human interpretative capacity, creating opportunities for AI-supported personalised risk assessment and treatment planning [11,12].

Despite encouraging findings, most studies remain retrospective and involve relatively small datasets, limiting external validity and delaying routine clinical implementation.

Clinical Decision Support Systems

Clinical decision support systems represent another important application of AI within paediatric allergy.

AI-assisted image recognition technologies have been investigated for differentiating allergic skin disease from common paediatric mimics, including viral exanthems and inflammatory dermatoses [11]. Such tools may prove particularly valuable in emergency departments and primary care settings where specialist allergy expertise is not immediately available.

Machine learning has also demonstrated potential within drug allergy assessment. Incorrect antibiotic allergy labels remain common in paediatric populations and contribute to broader-spectrum antibiotic prescribing, increased healthcare costs, and antimicrobial resistance. AI-assisted risk stratification may support identification of low-risk patients suitable for de-labelling pathways and improve antimicrobial stewardship [11].

Additionally, AI-integrated prescribing systems may have demonstrated some potential in reducing medication errors and identifying allergen-related prescribing risks [10]. However, their effectiveness depends on accurate and complete electronic health record documentation, and evidence demonstrating reductions in medication errors or adverse drug events remains limited [11].

Digital Health, Generative AI and Precision Allergy Medicine

Digital health technologies have become increasingly integrated within allergy care. Mobile applications now support symptom monitoring, medication adherence, allergen avoidance advice, and patient education [11].

More recently, advances in generative AI and large language models have introduced additional opportunities. These technologies may support patient education, clinical documentation, referral triage, healthcare communication, and administrative efficiency [13]. Within overstretched healthcare systems, such tools may help improve access to specialist information while reducing clinician workload.

Future applications may extend beyond traditional diagnostic support towards precision allergy medicine. Integration of clinical data with molecular diagnostics, environmental exposures, genomic information, and longitudinal patient-reported outcomes may facilitate increasingly personalised prediction models and treatment strategies [12,14]. Such approaches have the potential to move allergy care beyond simple diagnostic classification towards dynamic risk prediction and tailored treatment pathways.

A summary table mapping AI methodology to paediatric allergy targets is illustrated in Table 1.

**Table 1:** Applications of artificial intelligence methodologies in paediatric allergy

AI methodology	How it works	Current paediatric allergy applications	Potential clinical benefit	Current limitations
Machine learning (ML)	Learns patterns from structured clinical data to generate predictions or classifications.	Prediction of oral food challenge outcomes; food allergy diagnosis; anaphylaxis risk stratification; prediction of treatment response.	Improves diagnostic accuracy, reduces unnecessary oral food challenges, supports personalised risk prediction.	Predominantly retrospective studies, small datasets, limited external validation, geographic bias.
Deep learning (DL)	Uses multi-layer neural networks to analyse complex, high-dimensional data, particularly images and large datasets.	Automated interpretation of dermatological images (eczema, urticaria), pattern recognition from molecular allergology datasets, future wearable sensor analysis.	May improve diagnostic accuracy for skin manifestations and identify complex biomarker patterns beyond clinician interpretation.	Requires very large annotated datasets, limited explainability ("black-box"), few paediatric-specific validation studies.
Natural language processing (NLP)	Extracts clinically meaningful information from unstructured free-text data such as clinic letters and electronic health records.	Identification of allergy documentation, referral triage, extraction of allergy histories, support for drug allergy de-labelling pathways, automated clinical documentation.	Reduces administrative burden, improves documentation quality, facilitates clinical decision support.	Dependent on data quality, language variability, inconsistent clinical documentation, privacy concerns.
Clinical decision support systems (CDSS)	Integrates AI predictions into clinician workflows to provide real-time recommendations during patient care.	Risk stratification, prescribing support, allergen-related prescribing alerts, referral prioritisation, clinical pathway standardisation.	Supports evidence-based decision-making, improves workflow efficiency, may reduce inappropriate referrals.	Effectiveness depends on accurate EHR data, alert fatigue, workflow integration challenges, limited evidence for improved patient outcomes.
Generative AI and large language models (LLMs)	Generates human-like text and summarises information using large language models trained on extensive datasets.	Patient education, clinic letters, discharge summaries, referral drafting, clinical documentation, healthcare communication.	Improves administrative efficiency, enhances patient education, supports clinician productivity.	Risk of hallucinations, inaccurate medical advice, lack of transparency, requires clinician oversight and regulatory safeguards.
Future multi-modal AI models	Integrates multiple data sources including clinical history, laboratory biomarkers, genomics, environmental exposures, wearable devices and patient-reported outcomes.	Precision allergy medicine, dynamic risk prediction, prediction of disease progression, personalised treatment planning ("digital allergy twin").	Enables proactive, personalised allergy care and longitudinal disease monitoring.	Currently theoretical; requires prospective validation, interoperable healthcare systems, robust governance and equitable access

Discussion: current challenges and future directions

Over the past decade, AI has evolved from a theoretical concept to a potentially transformative tool within paediatric allergy. Unlike many other specialities, allergy medicine generates large volumes of multidimensional data encompassing clinical histories, laboratory biomarkers, molecular allergology profiles, environmental exposures, and longitudinal symptom patterns. Consequently, paediatric allergy is particularly well positioned to benefit from AI-driven approaches [6,11].

The most compelling evidence currently relates to food allergy diagnosis. Traditional investigations frequently struggle to distinguish clinically significant allergy from asymptomatic sensitisation, resulting in overdiagnosis and unnecessary dietary restriction [3,4]. Machine learning systems capable of integrating multiple variables simultaneously may provide a more nuanced assessment of allergy risk than isolated biomarkers alone. Importantly, the value of these systems may extend beyond improved diagnostic accuracy to the reduction of unnecessary oral food challenges and more efficient use of specialist resources [5,9,10].

A notable trend within the literature is the movement from diagnostic prediction towards precision medicine. Emerging AI models seek not only to determine whether allergy exists but also to predict disease trajectory, reaction severity, treatment response, and long-term outcomes. This approach aligns with broader developments across medicine and may be particularly relevant in paediatric populations, where allergic disease frequently evolves throughout childhood [14,15].

The emergence of generative AI and large language models represents another significant development. These technologies have potential applications in patient education, clinical documentation, referral management, and healthcare communication [11,12]. However, concerns regarding inaccurate outputs, misinformation, and lack of clinical oversight remain substantial. Within paediatric allergy, where errors may result in delayed recognition of anaphylaxis or inappropriate dietary advice, human supervision will remain essential.

Despite considerable promise, several barriers continue to limit the implementation of AI within paediatric allergy.

First, the majority of published studies remain retrospective and single-centre, with limited external validation. Algorithms developed using narrowly selected populations may perform less effectively when applied to diverse patient groups and healthcare systems.

Second, algorithmic transparency remains a major challenge. Many machine learning systems function as “black-box” models, generating predictions without readily explainable reasoning. Whilst acceptable in some research contexts, explainability is crucial within paediatric medicine, where clinical decisions frequently require detailed discussion with families and shared decision-making.

Third, digital inequality represents an important concern. Unequal access to technology and specialist services may result in AI disproportionately benefiting already advantaged populations, potentially widening existing healthcare disparities [14].

Fourth, most of the published studies originated from high-income Western countries, which may limit the generalisability of current algorithms to more diverse populations and lower-resource healthcare settings [11].

A particularly intriguing future direction is the development of a “digital allergy twin”. Inspired by emerging concepts in precision medicine, future AI systems may continuously integrate information from electronic health records, molecular allergology, environmental allergen exposures, wearable sensors, dietary records, and patient-reported outcomes to generate a dynamic digital representation of an individual child’s allergic disease. Rather than relying solely on episodic clinic encounters, these models could potentially predict periods of heightened anaphylaxis risk, identify patients most likely to respond to specific interventions, and provide personalised recommendations that evolve over time. Although currently theoretical, this concept represents a potential shift from reactive allergy management towards proactive and predictive care [11-15].

Importantly, the greatest impact of AI within paediatric allergy may not arise from superior diagnostic accuracy alone. Many healthcare systems continue to face rising allergy referral rates alongside shortages of specialist clinicians. Consequently, AI-assisted triage systems, referral prioritisation, clinic documentation, and patient education tools may ultimately deliver greater population-level benefits than diagnostic algorithms themselves. In this context, AI should be viewed not as a replacement for specialist clinicians but as a force multiplier capable of improving efficiency, expanding access to expertise, and supporting increasingly complex allergy services [11,13,14].

Perhaps the most important observation from the current literature is that future success should not be measured solely through improvements in predictive accuracy. While many studies report favourable performance metrics, few have demonstrated meaningful improvements in patient-centred outcomes. Future research should therefore prioritise prospective implementation studies evaluating effects on diagnostic delay, oral food challenge utilisation, quality of life, healthcare costs, emergency presentations, and clinician workload.

Generative AI and large language models may support patient education, clinical documentation and referral triage within paediatric allergy, but their implementation requires robust safety guardrails. These should include clinician oversight, human-in-the-loop review of AI-generated advice, prospective validation in real-world paediatric allergy settings, transparent and explainable outputs, and clear accountability for clinical decisions [12,13,15]. This is particularly important where inaccurate advice could result in missed anaphylaxis triggers, inappropriate dietary restriction, or unsafe drug allergy recommendations. Before routine clinical implementation, such tools should be evaluated within recognised regulatory and evidence frameworks, including digital health technology standards, software-as-a-medical-device regulation, and emerging high-risk AI governance frameworks [14,15].

Ultimately, AI is unlikely to replace clinicians in paediatric allergy. Instead, its greatest value will likely be as an adjunctive tool that reduces uncertainty, supports clinical decision-making, and enables increasingly personalised approaches to care. The future success of AI in paediatric allergy will likely be determined not by its ability to outperform clinicians, but by its ability to augment clinical expertise, reduce healthcare inequities, and facilitate a transition towards truly personalised allergy care.

A broader examination of the literature suggests that the increasing application of AI within allergy research reflects wider developments across healthcare. In their bibliometric analysis, Kong et al. demonstrated a substantial increase in machine learning publications within allergy research over the past decade, with particularly rapid growth following 2018 as computational methods became more accessible and larger clinical datasets became available [16]. Their findings identified food allergy, asthma, allergic rhinitis and precision medicine as the dominant research themes, highlighting a shift away from traditional hypothesis-driven analyses towards data-driven approaches capable of identifying complex relationships within heterogeneous patient populations. This trend is particularly relevant to paediatric allergy, where disease expression is influenced by dynamic interactions between genetic susceptibility, environmental exposures and immunological development throughout childhood.

The unique characteristics of paediatric allergic disease may make the speciality especially suited to AI-based approaches. Gutowska-Ślesik et al. argue that paediatric allergy generates large volumes of multidimensional data, including clinical histories, laboratory biomarkers, component-resolved diagnostics, environmental exposures and longitudinal outcome measures, creating an ideal environment for machine learning applications [17]. Unlike traditional statistical models that often evaluate variables independently, AI systems can simultaneously integrate multiple data streams to generate personalised predictions. Consequently, the potential value of AI extends beyond diagnostic support towards prediction of disease progression, treatment response and long-term clinical outcomes. Such capabilities align closely with the broader movement towards precision medicine, in which management strategies are increasingly tailored to individual patient characteristics rather than broad diagnostic categories.

Another important observation emerging from recent reviews is the expanding scope of AI applications within allergology. While early research predominantly focused on diagnostic prediction, contemporary work increasingly encompasses risk stratification, clinical decision support, healthcare workflow optimisation and patient engagement. Seurig et al. describe how advances in machine learning, natural language processing and predictive analytics are facilitating a transition from reactive models of allergy care towards more proactive and preventative approaches [18]. This evolution is particularly relevant in paediatric practice, where earlier identification of children at risk of severe allergic disease could allow targeted intervention before the development of significant morbidity. Furthermore, the integration of molecular allergology, genomics and environmental data may enable increasingly sophisticated models capable of identifying clinically meaningful disease endotypes and informing personalised therapeutic strategies.

Collectively, these reviews suggest that the future impact of AI in paediatric allergy may ultimately depend less on achieving marginal improvements in diagnostic accuracy and more on its ability to support system-wide transformation of allergy services [16-18]. Rising allergy prevalence, increasing referral volumes and persistent shortages of specialist clinicians have created significant pressures on healthcare systems internationally. In this context, AI-assisted triage, referral prioritisation, documentation support and patient education tools may provide substantial population-level benefits. However, all three reviews emphasise that successful implementation will require robust prospective validation, transparent algorithm development and careful consideration of ethical and regulatory challenges to ensure that technological innovation translates into meaningful improvements in patient care [16-18].

To enhance accessibility for both clinical and non-clinical audiences, a lay summary of the review is provided, while the principal clinical and research implications are summarised as key take-home messages.

Lay summary

Allergic diseases such as food allergy, eczema, asthma and hay fever are becoming increasingly common in children worldwide. Diagnosing and managing these conditions can be challenging because tests do not always accurately predict whether a child will develop symptoms, and specialist allergy services are often overstretched. AI refers to computer systems that can analyse large amounts of information and identify patterns that may not be obvious to clinicians. This review examined how AI has been used in paediatric allergy research between 2015 and 2025. Current evidence suggests that AI may help improve the diagnosis of food allergies, predict the risk of severe allergic reactions, and support doctors in making clinical decisions. AI may also assist with interpreting complex allergy test results, prioritising referrals, reducing unnecessary investigations, and improving access to allergy care. Recent advances in digital health technologies and generative AI have created further opportunities to support patient education, symptom monitoring, clinical documentation and healthcare communication. In the future, AI could contribute to more personalised allergy care by combining information from medical records, allergy tests, environmental exposures and patient-reported symptoms to help predict disease progression and guide treatment decisions. Although the results are promising, most studies remain at an early stage. Further research is needed to ensure that AI systems are accurate, safe, transparent and effective before they can be routinely used in clinical practice. 

Take-home messages

AI has demonstrated considerable potential to enhance the diagnosis and risk prediction of paediatric allergic diseases, particularly in areas such as food allergy and anaphylaxis. Beyond diagnostic applications, AI is increasingly being integrated into clinical decision support systems, digital health platforms, patient education resources, referral triage pathways, and healthcare workflow optimisation, reflecting its growing role across the wider allergy care pathway. However, despite these promising developments, most evidence remains at an early stage, and robust prospective validation is still required. Careful consideration of ethical, regulatory, and equity-related challenges will also be essential to ensure that AI technologies are implemented safely, effectively, and equitably in routine clinical practice.

## Conclusions

AI represents a promising and rapidly evolving field within paediatric allergy. Advances in machine learning, clinical decision support systems, digital health technologies, and generative AI have demonstrated potential to improve diagnostic accuracy, risk stratification, healthcare efficiency, and patient engagement. Although current evidence is encouraging, most applications remain in early developmental stages. Future progress will require multicentre prospective studies, externally validated paediatric datasets, transparent algorithms, and robust regulatory oversight. With appropriate validation and governance, AI may become an important adjunct to clinicians and contribute to safer, more efficient, and increasingly personalised paediatric allergy care.

## References

[1] Savage J, Johns CB (2015). Food allergy: epidemiology and natural history. Immunol Allergy Clin North Am.

[2] McWilliam V, Koplin J, Lodge C, Tang M, Dharmage S, Allen K (2015). The prevalence of tree nut allergy: a systematic review. Curr Allergy Asthma Rep.

[3] Chafen JJ, Newberry SJ, Riedl MA (2010). Diagnosing and managing common food allergies: a systematic review. JAMA.

[4] Sicherer SH, Sampson HA (2018). Food allergy: a review and update on epidemiology, pathogenesis, diagnosis, prevention, and management. J Allergy Clin Immunol.

[5] Greenhawt M, Shaker M, Wang J (2020). Peanut allergy diagnosis: a 2020 practice parameter update, systematic review, and GRADE analysis. J Allergy Clin Immunol.

[6] Deo RC (2015). Machine learning in medicine. Circulation.

[7] Topol EJ (2019). High-performance medicine: the convergence of human and artificial intelligence. Nat Med.

[8] Rajkomar A, Dean J, Kohane I (2019). Machine learning in medicine. N Engl J Med.

[9] Gryak J, Georgievska A, Zhang J, Najarian K, Ravikumar R, Sanders G, Schuler CF 4th (2024). Prediction of pediatric peanut oral food challenge outcomes using machine learning. J Allergy Clin Immunol Glob.

[10] Patel SJ, Chamberlain DB, Chamberlain JM (2018). A machine learning approach to predicting need for hospitalization for pediatric asthma exacerbation at the time of emergency department triage. Acad Emerg Med.

[11] van Breugel M, Greenhawt M, Eguiluz-Gracia I, Torres Jaén MJ, Anagnostou A, Koppelman GH (2026). Artificial intelligence in allergy and immunology: Recent developments, implementation challenges, and the road toward clinical impact. J Allergy Clin Immunol.

[12] Konstantinou G, Portnoy J (2025). Artificial intelligence and the future of allergy practice. J Allergy Clin Immunol Pract.

[13] Turner PJ, Worm M, Ansotegui IJ (2019). Time to revisit the definition and clinical criteria for anaphylaxis?. World Allergy Organ J.

[14] Wong DS, Santos AF (2024). The future of food allergy diagnosis. Front Allergy.

[15] Xiao N, Huang X, Wu Y (2025). Opportunities and challenges with artificial intelligence in allergy and immunology: a bibliometric study. Front Med (Lausanne).

[16] Kong E, Cucco A, Custovic A, Fontanella S (2026). Machine learning in allergy research: a bibliometric review. Immunol Lett.

[17] Gutowska-Ślesik J, Samoliński B, Krzych-Fałta E (2023). The increase in allergic conditions based on a review of literature. Postepy Dermatol Alergol.

[18] Seurig S, Traidl S, Mathes S (2026). The role of artificial intelligence in modern allergology: a review of applications in diagnosis, prediction, and management. JEADV Clinical Practice.

